# Safety first: evidence for delay of radical prostatectomy without use of androgen deprivation therapy during COVID-19

**DOI:** 10.2217/fon-2020-0388

**Published:** 2020-05-14

**Authors:** Xuan Rui Sean Ong, Benjamin Condon, Dominic Bagguley, Nathan Lawrentschuk, Arun Azad, Declan Murphy

**Affiliations:** ^1^Division of Cancer Surgery, Peter MacCallum Cancer Centre, Parkville, Victoria, Australia; ^2^ EJ Whitten Foundation Prostate Cancer Research Centre, Epworth Health, Victoria, Australia

**Keywords:** ADT, androgen deprivation therapy, cancer, COVID-19, hormonal therapy, prostate, prostatectomy, surgery, urologic

The global coronavirus (COVID-19) pandemic presents a unique situation whereby governing bodies seek to balance the risks of deferring treatment with the preservation of hospital resources. Like most of the world, elective surgery for certain cancers have come to a virtual standstill in preparation for the potential need for emergency beds and equipment. The treatment for prostate cancer has been one of the fields that has seen a large amount of change to its triage system. For the moment, there are no widely accepted ‘COVID-19 era’ ;management guidelines for patients with prostate cancer. Hospitals have formed guidelines which take into account their own resources and availabilities. Many difficult decisions need to be made on a case by case basis, bearing in mind hospital capabilities, disease risk, patient factors and of course patients’ ;mental health and anxiety.

Radical prostatectomy is usually reserved for localized prostate cancer. It requires the use of an anaesthetic machine which contains a ventilator, personal protective equipment, hospital beds and healthcare workers. Furthermore, the patient will also use a hospital room postoperatively and in the worst circumstance may even need ICU admission. All relevant considerations in these times. Understandably, most hospitals have decreased the number of radical prostatectomies performed every week. This has made triaging particularly difficult given its high demand. Here we review the evidence for the safety of delayed radical prostatectomies and explore the role of androgen deprivation therapy (ADT) in this unprecedented situation.

Delayed treatment of favorable risk prostate cancer has been a widely discussed topic in urology during the rise of active surveillance over the last decade. Research has centered around balancing the high rates of adverse outcomes from treatment and the potential risk of progression of disease.

Several studies have shown that low risk prostate cancer can be safely monitored without intervention which should continue even during the COVID-19 pandemic. The PRIAS group recruited 5302 men across 18 countries to be included in their prospective active surveillance study. Initially, only low risk disease was included (Gleason score 3+3, stage not higher than cT2c, PSA 10 ;ng/ml, two or fewer cores positive for PCa, PSA density 0.2 ;ng/ml per cubic centimetre), but from 2012 favorable intermediate risk disease (Gleason score 3+4 [<10%], stage not higher than cT2c, PSA 10 ;ng/ml, two or fewer cores positive for PCa, PSA density 0.2 ;ng/ml per cubic centimetre) was added to the analysis. A 10-year follow-up study published in 2016 found that prostate specific mortality rates of all men was <1% [1]. Klotz *et al.* showed a 1.5% rate of prostate cancer specific death and 2.8% rate of developing metastatic disease in a Toronto study of 819 men with low risk disease on active surveillance at 15 ;years of follow-up [2]. Tosoian *et al.* studied 1298 men with very low and low risk disease (clinical stage ;≤T2a, PSA <10 ;ng/ml, and Gleason score ≤6). These authors found cancer specific survival and metastatic free survival rates of 99.9% and 99.4% at 15 ;years of active surveillance [3]. Overall this is compelling evidence demonstrating the safety of long-term active surveillance for very low, low and favorable intermediate risk prostate cancer.

Localized unfavorable intermediate risk and high risk cancers pose a different challenge. If the patient has more than 10 ;years life expectancy, then studies have shown benefits in mortality when receiving either radical prostatectomy or radiation therapy and ADT compared with watchful waiting [4,5]. In normal circumstances, high volumes of patients are already on a radical prostatectomy waiting list. Median wait times have been reported in some centers at 91 ;days [6]. COVID-19 will force waiting lists to become more congested. Fortunately, being an indolent disease, prolonged delays in surgery can be carried out safely.

Fossati *et al.* studied 4156 men who received a radical prostatectomy from 2006–2012. They looked for a correlation between time from prostate cancer diagnosis to surgical treatment and postoperative biochemical recurrence (BCR) and clinical recurrence. Of the patients studied, 35%, 50%, and 15% of patients were affected by low, intermediate and high-risk prostate cancer respectively. The authors found that time from diagnosis to treatment was significantly associated with an increased risk of BCR and clinical recurrence in high risk patients only. They were able to estimate that the risk of BCR and CR only becomes clinically significant at around 12 ;months after diagnosis [7]. Of note, Boorjian *et al.* found similar results. Their study of 3149 men found that time to radical prostatectomy was not a predictor of BCR even in the high risk group of up to 12 ;months [8]. O’Callaghan *et al.* also found no significant association with delay to treatment and prostate cancer specific mortality when analysing all risk groups [9]. Other reports have found results both in line with the above [10–15] or contradicting these results [16]. However, these studies were either only analysed one category of risk or split time as a dichotomous variable (ie., <31 ;days compared with >70 ;days, <60 ;days compared with >60 ;days, <2.5 ;months or >2.5 ;months and <6 ;months compared with >6 ;months). Despite the delay in treatment in the studies above, ADT was not utilized in these cohorts to stop disease progression.

Despite having a role in radiotherapy, neoadjuvant ADT is not recommended in guidelines for patients undergoing radical prostatectomy [17]. A systematic review by Kumar *et al.* found that neoadjuvant ADT for radical prostatectomy did show significant reduction in positive margin rates, lymph node involvement, pathological staging and organ confined rates but did not improve overall survival and disease free survival [18]. Moreover, treatment with ADT substantially increases risk of cardiovascular, metabolic, musculoskeletal, neurological, reproductive and psychological conditions [19].

As communities around the world work to ‘flatten the curve’ ;of the deadly COVID-19, many groups are also dedicated to planning the pathway out. For prostate cancer, urology teams will need to manage an already overcrowded list of patients needing radical prostatectomies. Healthcare workers will be working hard to efficiently prioritize the outstanding cases. Some difficult conversations may need to be had. Fortunately, the literature demonstrates that delays of up to 12 ;months with no ADT are not associated with adverse outcomes postoperatively for all categories of localized prostate cancer. The recently published European Association of Urology guidelines reflect this by recommending postponement of radical prostatectomy until after the pandemic without use of ADT [20]. We remain optimistic about gaining ascendancy against COVID-19 and working toward the timely management of prostate cancer.

## References

[1] Bokhorst LP, Valdagni R, Rannikko A A decade of active surveillance in the PRIAS study: an update and evaluation of the criteria used to recommend a switch to active treatment. Eur. Urol. 70, 954–960 (2016). 27329565 10.1016/j.eururo.2016.06.007

[2] Klotz L, Vesprini D, Sethukavalan P Long-term follow-up of a large active surveillance cohort of patients with prostate cancer. J. Clin. Oncol. 33, 272–277 (2015). 25512465 10.1200/JCO.2014.55.1192

[3] Tosoian JJ, Mamawala M, Epstein JI Intermediate and longer-term outcomes from a prospective active-surveillance program for favorable-risk prostate cancer. J. Clin. Oncol. 33, 3379 (2015). 26324359 10.1200/JCO.2015.62.5764 PMC4863946

[4] Bill-Axelson A, Holmberg L, Filén F Radical prostatectomy versus watchful waiting in localized prostate cancer: the Scandinavian prostate cancer group-4 randomized trial. J. Natl Cancer Inst. 100, 1144–1154 (2008). 18695132 10.1093/jnci/djn255 PMC2518167

[5] Wilt TJ, Brawer MK, Barry MJ The prostate cancer intervention versus observation trial:VA/NCI/AHRQ Cooperative Studies Program #407 (PIVOT): design and baseline results of a randomized controlled trial comparing radical prostatectomy to watchful waiting for men with clinically localized prostate cancer. Contemp. Clin. Trials. 30, 81–87 (2009). 18783735 10.1016/j.cct.2008.08.002

[6] Siemens DR, Schulze KM, Mackillop WJ, Brundage MD, Groome PA A population-based study of the waiting times for prostatectomy in Ontario. Can. J. Urol. 12, 2568–2574 (2005). 15877937

[7] Fossati N, Rossi MS, Cucchiara V Evaluating the effect of time from prostate cancer diagnosis to radical prostatectomy on cancer control: can surgery be postponed safely? Urol. Oncol. Semin. Orig. Investig. 35, 150 (2017). 10.1016/j.urolonc.2016.11.010 27986374

[8] Boorjian SA, Bianco FJ, Scardino PT, Eastham JA Does the time from biopsy to surgery affect biochemical recurrence after radical prostatectomy? BJU Int. 96, 773–776 (2005). 16153197 10.1111/j.1464-410X.2005.05763.x

[9] O'Callaghan ME, Shi Z, Kopsaftis T, Moretti K Prostate cancer outcomes and delays in care. Int. Urol. Nephrol. 49, 449–455 (2017). 28083860 10.1007/s11255-017-1508-z

[10] Korets R, Seager CM, Pitman MS, Hruby GW, Benson MC, McKiernan JM Effect of delaying surgery on radical prostatectomy outcomes: a contemporary analysis. BJU Int. 110, 211–216 (2012). 22093486 10.1111/j.1464-410X.2011.10666.x

[11] Holmström B, Holmberg E, Egevad L Outcome of primary versus deferred radical prostatectomy in the national prostate cancer register of Sweden follow-up study. J. Urol. 184, 1322–1327 (2010). 20723940 10.1016/j.juro.2010.06.008

[12] Vickers AJ, Bianco FJ, Boorjian S, Scardino PT, Eastham JA Does a delay between diagnosis and radical prostatectomy increase the risk of disease recurrence? Cancer: Interdisciplinary International Journal of the American Cancer Society. 106, 576–580 (2006). 10.1002/cncr.21643 PMC1774862 16353213

[13] Graefen M, Walz J, Chun KHF, Schlomm T, Haese A, Huland H Reasonable delay of surgical treatment in men with localized prostate cancer – impact on prognosis? Eur. Urol. 47, 756–760 (2005). 15925069 10.1016/j.eururo.2005.02.004

[14] Shibata A, Mohanasundaram UM, Terris MK Interval from prostate biopsy to radical prostatectomy: effect on PSA, gleason sum, and risk of recurrence. Urology. 66, 808–813 (2005). 16230143 10.1016/j.urology.2005.04.069

[15] Khan MA, Mangold LA, Epstein JI, Boitnott JK, Walsh PC, Partin AW Impact of surgical delay on long-term cancer control for clinically localized prostate cancer. J. Urol. 172, 1835–1839 (2004). 15540733 10.1097/01.ju.0000140277.08623.13

[16] O'Brien D, Loeb S, Carvalhal GF Delay of surgery in men with low risk prostate cancer. J. Urol. 185, 2143–2147 (2011). 21496847 10.1016/j.juro.2011.02.009

[17] Mottet N, van den B RCN, Briers E EAU-EANM-ESTRO-ESUR-SIOG guidelines on prostate cancer 2019. Eur. Assoc. Urol. Guidel. 2019. 53, 1–161 (2019).

[18] Kumar S, Shelley M, Harrison C, Coles B, Wilt TJ, Mason MD Neo-adjuvant and adjuvant hormone therapy for localized and locally advanced prostate cancer. Cochrane Database Syst. Rev. 4, (2006). 10.1002/14651858.CD006019.pub2 PMC8996243 17054269

[19] Edmunds K, Tuffaha H, Galvão DA, Scuffham P, Newton RU Incidence of the adverse effects of androgen deprivation therapy for prostate cancer: a systematic literature review. Support. Care Cancer. 1–15 (2020). 31912360 10.1007/s00520-019-05255-5

[20] European Association of Urology. COVID-19 combined oncology recommendations (2020). https://uroweb.org/wp-content/uploads/Combined-oncology-COVID-19-recommendations.pdf

